# Antithrombin effect on coagulation and fibrinolytic profiles after living donor liver transplantation: a pilot study

**DOI:** 10.1111/j.1751-553X.2007.01008.x

**Published:** 2009-02

**Authors:** J KANEKO, Y SUGAWARA, S TAMURA, J TOGASHI, Y MATSUI, M MAKUUCHI

**Affiliations:** Artificial Organ and Transplantation Division, Department of Surgery, Graduate School of Medicine, University of TokyoTokyo, Japan

**Keywords:** Liver transplantation, coagulation, heparin, antithrombin, heparin, thrombin

## Abstract

Early after liver transplantation, patients are in a hypercoagulable state because of an imbalance between coagulation and fibrinolysis because of the slow recovery of depleted anticoagulant proteins. Antithrombin (AT) is used in anticoagulant protocols to prevent thrombosis. The subjects of the present study were 17 men and eight women that underwent living donor liver transplantation. The initial 15 cases were administered AT concentrate (1500 U/day) on postoperative days (POD) 1 through 3 (AT group) and the following 10 consecutive cases were not administered AT (control). AT, thrombin-AT complex, plasmin-alpha2 plasmin inhibitor complex, thrombomodulin, fibrin degradation product D-dimer (FDP-DD) level, prothrombin time international normalized ratio, activated partial thromboplastin time, and platelet counts were measured. In the AT group, AT activity was maintained at levels >80% for 5 days after transplantation. In the control group, AT activity did not return to normal during the first 2 weeks after the operation. FDP-DD levels were significantly higher in the control group than in the AT group (*P* < 0.05). Six patients in the control group and three patients in the AT group required transfusions with platelet concentrate (*P* < 0.05). AT supplementation might reduce FDP-DD levels and prevent decreased platelet counts in the early stages after liver transplantation.

## Introduction

Antithrombin (AT) is a 58-kDa plasma glycoprotein synthesized in the liver that acts as a serine protease inhibitor affecting multiple components of the intrinsic, extrinsic, and common coagulation pathways (Quinsey *et al.*, 2004). Palareti *et al.* (1991) reported that AT levels are stable during liver transplantation. In their study, AT levels after surgery were approximately 80% that of the preoperative level when the AT concentrate was not administered. During surgery, Palareti *et al.* (1991) transfused an average of 8150 ml fresh frozen plasma, which might have affected AT levels, because fresh frozen plasma contains high levels of AT. Stahl *et al.* (1990), however, reported that the hypercoagulable state persists during the early period after deceased donor liver transplantation in cirrhotic patients. There is an imbalance between coagulation and fibrinolysis because the levels of anticoagulant proteins, such as proteins C, S, and AT return to normal slowly. Hashikura *et al.* (1995)advocated that AT should be included in the anticoagulant protocol during the post-transplant course for pediatric patients.

A detailed discussion of whether the administration of AT concentrates is truly necessary after liver transplantation has not yet been presented. The purpose of this pilot study was to clarify the feasibility of using AT concentrates in an anticoagulant protocol in the early stages after liver transplantation.

## Materials and methods

The subjects were 17 men and eight women (age range: 19–67 years; median 51 years) that underwent living donor liver transplantation at the University of Tokyo Hospital from July 2005 to April 2006.

### Administration of antithrombin concentrates

This pilot study protocol was approved by the University of Tokyo institutional review board. Informed consent was obtained from all participating patients. AT concentrates (1500 U/day, Anthrobin P; ZLB Behring K.K., Tokyo, Japan) were administered to the initial 15 cases on postoperative days (POD) 1 through 3 (AT group) and no AT was administered in the next 10 consecutive cases (control group).

The indications for living donor liver transplantation included hepatitis B or C-related cirrhosis (AT group, *n* = 9; control, *n* = 6), cholestatic liver diseases (AT, *n* = 4; control, *n* = 2), fulminant hepatic failure (AT, *n* = 1; control, *n* = 1), alcoholic liver cirrhosis (control, *n* = 1), and others (AT, *n* = 1). The types of grafts used were right liver (AT group, *n* = 8; control group, *n* = 8), left liver with caudate lobe (AT, *n* = 7; control, *n* = 1), and right lateral sector (control, *n* = 1).

### Anticoagulant therapy protocol

Except for the AT, both groups were treated with the same anticoagulant therapy (Kaneko *et al.*, 2005). The anticoagulant therapy consisted of heparin (unfractionated heparin sodium, UFH), dalteparin, prostaglandin E1, and a protease inhibitor. Initially, we started dalteparin (25 IU/kg/day) on POD 1, then switched to UFH (5000 U/day) on POD 2 and 3 because there are fewer hemorrhagic complications in patients receiving dalteparin than in those receiving UFH (Dunn & Jarvis, 2000). The dose of UFH was changed according to the activated clotting time. The agents were administered by continuous intravenous infusion. The targeted activated clotting time ranged between 130 and 160 s. This range corresponded to the upper half of the normal range of an FTCA510 test tube (normal range 105–167 s) using a HEMOCRON Response or Model 401 (International Technodyne Corporation, Lyndhurst, NJ, USA). Prostaglandin E1 (0.01 μg/kg/h) and a protease inhibitor (mesilate gabexate; 1 mg/kg/h) were administered intravenously just after the operation for 3 days. When the platelet count was <30^9^/l, patients were transfused with platelet concentrate. If a patient had more than one liter of ascites drained, several units of fresh frozen plasma were transfused after liver transplantation.

### Monitoring the coagulable and fibrinolytic parameters

Antithrombin (TESTZYM S AT; Daiichi Pure Chemicals, Tokyo, Japan), thrombomodulin (PANACELA PLATE; DAIICHI Fine Chemical, Toyama, Japan), plasmin-alpha2 plasmin inhibitor complex (LPIA Ace PPI; Mitsubishi Kagaku Iatron, Tokyo, Japan), thrombin-antithrombin complex (SRL, Tokyo, Japan), and fibrin degradation product D-dimer (FDP-DD, LPIA Ace d-Dimer; Mitsubishi Kagaku Iatron) were measured the day before the operation and on POD 1, 2, 3, 5, 7, 10, and 14. Complete blood count, prothrombin time international normalized ratio (INR), total bilirubin, aspartate aminotransferase, alanine aminotransferase, and creatinine were measured once or twice on successive days for 2 weeks after living donor liver transplantation.

### Statistical analysis

The time-series coagulable and fibrinolytic data were compared between the groups by two-way repeated measures analysis of variance. The number of patients transfused with platelet concentration and patient preoperative data in both groups were analyzed by Student’s *t*-test. In these analyses, a *P*-value of <0.05 was considered to be statistically significant. Data from blood samples taken after the operation were compared with preoperative data in each group by a multiple comparison using Bonferroni’s correction. The test was applied for AT, thrombin-antithrombin complex, PIC, TM, FDP-DD, INR, activated partial thromboplastin time (APTT), and platelet count, for which preoperative data were available. A *P*-value of <0.0083 was considered to be statistically significant. A statistical analysis software package (SPSS 11.0J, SPSS Japan Inc., Tokyo, Japan) was used for data analysis. Data are expressed as mean ± standard deviation.

## Results

**Figure 1:**
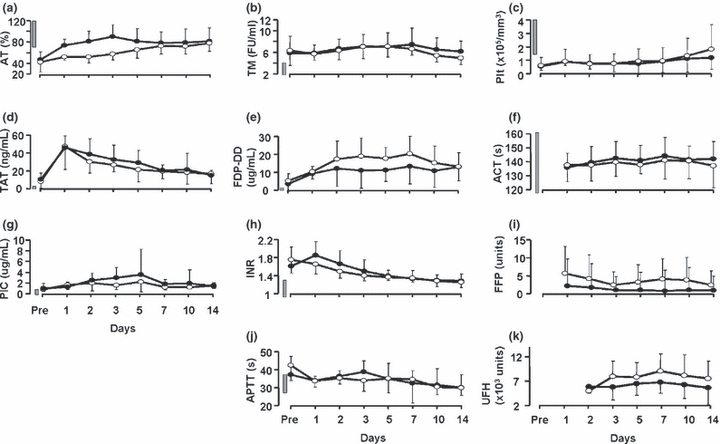
Changes in the levels of antithrombin (AT, a), thrombin-antithrombin complex (TAT, b), plasmin-alpha2 plasmin inhibitor complex (PIC, c), thrombomodulin (TM, d), fibrin degradation product d-dimer (FDP-DD, e), prothrombin time international normalized ratio (INR, f), activated partial thromboplastin time (APTT, g), platelet count (Plt, h), activated clotting time (ACT, i), units of fresh frozen plasma used (FFP, j), and dose of unfractionated heparin sodium used (UFH, k). Grey bars to the right of the y axis show the normal range. Closed and open circles are AT and control groups, respectively. The error bars represent standard deviation.

Patient profiles, including age, model for end stage liver disease score (Wiesner *et al.*, 2003), procured graft weight, and intraoperative use of fresh frozen plasma, were not different between groups (Table 1). In the AT group, AT activity was significantly increased beginning on POD 1 compared with the preoperative level and was maintained at >80% until POD 5. In the control group, AT activity gradually increased (Figure 1). There was a significant difference between groups (*P* = 0.03). The thrombin-AT complex levels were markedly elevated on POD 1 in both groups and then gradually decreased, although they did not return to the preoperative level during the observation period. Thrombin-AT complex levels in the AT group tended to be higher than those in the control group, although there was no significant difference between groups (*P* = 0.06).

**Table 1:** Patients profiles

Groups	AT (*n* = 15)	Control (*n* = 10)	*P*-value
Age	52 (23–64)	52 (18–65)	0.96
MELD score	13 (4–37)	18 (7–25)	0.18
Graft weight (g)	529 (348–681)	603 (434–757)	0.07
Fresh frozenplasma (l)*	3.9 (1.2–5.6)	3.7 (1.2–6.0)	0.7

MELD, model for end-stage liver disease.

* Volume during the operation.

The plasmin-alpha2 plasmin inhibitor complex (*P* = 0.30) or thrombomodulin (*P* = 0.88) levels did not significantly differ between groups. The FDP-DD levels peaked at POD 7 in both groups, but were significantly higher in the control group (*P* = 0.03). There was no inter-group difference in the INR (*P* = 0.40), APTT (*P* = 0.71), or platelet count (*P* = 0.54). Six patients in the control group and three patients in the AT group were transfused with platelet concentrate (*P* < 0.05).

There were no significant differences between groups in the other factors, including activated clotting time (*P* = 0.71), units of fresh frozen plasma (*P* = 0.16), UFH dose (*P* = 0.09), aspartate aminotransferase (*P* = 0.39), alanine aminotransferase (*P* = 0.61), total bilirubin (*P* = 0.34), or creatinine (*P* = 0.59). None of the patients experienced complications such as vascular thrombosis or hemorrhage during the observation period.

## Discussion

Stahl *et al.* (1990) reported that mean AT activity levels in whole liver transplant recipients are 53% and 67% on POD 3 and 5, respectively. In the control group in our study, AT activity did not reach normal levels during the first 2 weeks after the operation. This finding suggests that the recovery of AT levels after partial liver transplantation is slower than that after whole liver transplantation. One possible reason is that AT is produced by the liver (Bock *et al.*, 1982) and the level is regulated by the graft size (Kaneko *et al.*, 2005). Preoperative AT levels (Palareti *et al.*, 1991) and the time for AT levels to return to normal after transplantation (Stahl *et al.*, 1990) differ among the various indications for liver transplantation. The demographics of Stahl’s patients are similar to those of ours: 33% and 20% of the patients, respectively, had cholestatic liver disease.

In pediatric liver transplantation, AT levels during the postoperative course are seldom reported. Hashikura *et al.* (1995) suggested that AT levels should return to 80–100% of preoperative activity 2 weeks after surgery, based on the antithrombotic effects of heparin. Indeed, in their study, 2 weeks after surgery, the administration of AT concentrate increased AT levels to >80% of its initial activity. In our AT group, however, AT levels during the first 2 weeks after the transplantation were at least 10% lower than those in their study. The dose of AT concentrate administered in the present study was lower than that administered in Hashikura *et al.*‘s study to adjust for patient body weight, although the precise dose was not described in their report.

Hepatic artery thrombosis is more common in split, pediatric, or living donor liver transplantation (Heffron *et al.*, 2005) because of the small diameter of the anastomosed hepatic arteries. Transplant surgeons should carefully select the anticoagulant therapy for partial liver transplantation. An appropriate AT level for an adequate anticoagulant effect has been reported. Kauffmann *et al.* (1978) reported the AT level threshold with antithrombotic effects in patients with nephritic syndrome: eight of 11 patients with AT levels of <70% developed thrombosis, whereas only one of 37 patients with AT levels >70% experienced thrombosis. On the other hand, a >60% AT level is required for heparin to exert an anticoagulant effect (Francis *et al.*, 1991) to prevent deep vein thrombosis after orthotopic surgery. In our study, the doses of UFH and AT might be related. The dose of UFH used in the AT group tended to be higher than that used in the control group when activated clotting time was adjusted to our target level.

It has been controversial whether disseminated intravascular coagulation (DIC) occurs during deceased donor liver transplantation. Lewis *et al.* (1989) and Bakker *et al.* (1993) placed doubt on the existence of DIC during surgery because of the small decrease in coagulation factors and the lack of an increase in the levels of fibrin degradation products. A patient might be in DIC status postoperatively after liver transplantation. The diagnosis of DIC using a scoring system (Taylor *et al.*, 2001) is not suitable for post-transplant patients (Carr, McKinney & McDonagh, 1989), which makes it difficult to diagnose in this situation. The high FDP-DD levels and consumption of concentrated platelets in the control group support our hypothesis. Low FDP-DD levels and concentrated platelet consumption indicate that UFH with AT might be an effective therapeutic combination for DIC. Ischemia-reperfusion injury in the graft and DIC present with a high thrombomodulin level. AT has anti-inflammatory effects on both of these conditions (Sido *et al.*, 1995; Iba & Kidokoro, 2002), although there was no significant difference in the postoperative thrombomodulin levels between groups in the present study.

One of the limitations of this study is that our anticoagulant protocol is based on activated clotting time. According to a previous report (De Waele *et al.*, 2003), activated clotting time is less sensitive than APTT in low dose UFH (5000–10 000 U/day). Fluctuation of ACT in this study might also be due to the other factors than the dose of UFH, including platelet count and function (Moorehead, Westengard & Bull, 1984; Girardi, Sudi & Muntean, 2000). The appropriate target APTT level after liver transplantation has not been clarified. The findings of our study indicate that APTT level was maintained within the upper half of the normal range during the first week after transplantation. These data might be helpful for APTT-based anticoagulation therapy.

## Conclusion

Antithrombin administration early after partial liver transplantation might be important for securing an adequate anticoagulant effect. Although the results of this pilot study do not prove its clinical advantage, the combined use of AT and UFH might reduce FDP-DD levels as well as prevent a postoperative drop in the platelet count.
